# A Pilot Study on the Cutoff Value of Related Brain Metabolite in Chinese Elderly Patients With Mild Cognitive Impairment Using MRS

**DOI:** 10.3389/fnagi.2021.617611

**Published:** 2021-04-09

**Authors:** Lihua Zhao, Jinlong Teng, Wei Mai, Jiahui Su, Bihan Yu, Xiucheng Nong, Chong Li, Yichen Wei, Gaoxiong Duan, Xiangming Deng, Demao Deng, Shangjie Chen

**Affiliations:** ^1^Department of Acupuncture, First Affiliated Hospital, Guangxi University of Chinese Medicine, Nanning, China; ^2^Department of Radiology, First Affiliated Hospital, Guangxi University of Chinese Medicine, Nanning, China; ^3^Department of Rehabilitation, Bao'an Hospital, Southern Medical University, Shenzhen, China

**Keywords:** N-acetyl aspartate, creatine, choline hippocampus, posterior cingulate gyrus, mild cognitive impairment

## Abstract

**Objective:** This cross-sectional study aimed to distinguish patients with mild cognitive impairment (MCI) from patients with normal controls (NCs) by measuring the levels of *N*-acetyl aspartate (NAA), total creatinine (tCr), and choline (Cho) in their hippocampus (HIP) and their posterior cingulate gyrus (PCG) by using proton magnetic resonance spectroscopy (MRS) and to predict the cutoff value on the ratios of metabolites. We further aimed to provide a reference for the diagnosis of MCI in elderly patients in China.

**Methods:** About 69 patients who underwent a clinical diagnosis of the MCI group and 67 patients with NCs, the Mini-Mental Status Examination (MMSE) score, the Montreal Cognitive Assessment (MoCA) score, and MRS of the bilateral HIP and bilateral PCG were considered. The ratio of NAA/tCr and Cho/tCr in the bilateral HIP and bilateral PCG was calculated. The relationship between the ratios of metabolites and the scores of MMSE and MoCA was analyzed, and the possible brain metabolite cutoff point for the diagnosis of MCI was evaluated.

**Results:** Compared with the NC group, the scores of MMSE and MoCA in the MCI group decreased significantly (*p* < 0.05); the ratio of NAA/tCr in the bilateral HIP and bilateral PCG and the ratio of Cho/tCr at the right HIP in the MCI group decreased significantly (*p* < 0.05); however, there was no significant difference in the ratio of Cho/tCr in the left HIP and bilateral PCG between the two groups (*p* > 0.05). The correlation coefficient between MMSE/MoCA and the ratio of NAA/tCr was 0.49–0.56 in the bilateral HIP (*p* < 0.01). The best cutoff value of NAA/creatine (Cr) in the left HIP and the right HIP was 1.195 and 1.19. Sensitivity, specificity, and the Youden index (YDI) in the left HIP and the right HIP were (0.725, 0.803, 0.528) and (0.754, 0.803, 0.557), respectively.

**Conclusion:** The level of metabolites in the HIP and the PCG of patients with MCI and of those with normal subjects has a certain correlation with the score of their MMSE and MoCA. When the value of NAA/tCr in the left HIP and right HIP is <1.19, it suggests that MCI may have occurred. According to this cutoff point, elderly patients with MCI in China could be screened.

## Introduction

Alzheimer's disease (AD), the most common form of dementia, is increasing worldwide. According to the world Alzheimer's report (Patterson, 2018), there were about 50 million people suffering from dementia in the world in 2018, which is expected to more than double by 2050. However, there is no effective way to treat AD. In terms of drug treatment, the recent drugs for tau pathology and β-amyloid (Aβ) fail to treat the disease in the stage of mild or moderate dementia (Doody et al., 2014; Salloway et al., 2014). Mild cognitive impairment (MCI) is a kind of cognitive disorder syndrome, which is a transitional stage between normal aging and AD. The main clinical manifestations of MCI are the decline of memory functions and the degree of cognitive impairment not being consistent with the education level and age, but it has not reached the diagnosis of AD (Petersen, 2016). MCI, as a prophase of AD, provides an important opportunity for potential interventions to prevent AD (Petersen et al., 2014), so it is very important to diagnose MCI as early and accurately as possible.

At present, the diagnosis of MCI mainly depends on the clinical performance and rating scales, among which the Mini-Mental Status Examination (MMSE) and the Montreal Cognitive Assessment (MoCA) are common scales. The disadvantage of scale testing is that it is easy to be affected by age, cultural background, and education level and it shows human error in large. The levels of tau protein and of β-amyloid protein (Aβ) in the cerebrospinal fluid are used as diagnostic indexes of MCI (Handels et al., 2017). However, the detection of cerebrospinal fluid needs an invasive lumbar puncture, so it is not easy to be accepted by patients. With the continuous development of imaging technology, magnetic resonance spectroscopy (MRS) is the main non-invasive imaging technology used to study the local metabolism, biochemical changes, and quantitative analysis of compounds in living tissues and organs, which can detect the activity and function of brain neurons. In recent years, it has been widely used in the diagnosis and monitoring of neurodegenerative diseases (Graff-Radford and Kantarci, 2013; Öz et al., 2014). The hippocampus (HIP) plays an important role in memory processing. The posterior cingulate gyrus (PCG) is usually affected by neurodegenerative diseases. Therefore, many studies place a region of interest (ROI) in these two areas: *N*-acetyl aspartate (NAA) is an amino acid mainly existing in neurons, dendrites, and axons of the central nervous system, and it actively participates in the synthesis of myelin. Its diagnostic function is based on its ability to quantify the damage or loss of regional neurons. Therefore, it has been widely used as a marker of neuron density and neurometabolic adaptability in the study of MRS of neurodegenerative diseases (Mohamed et al., 2014). Choline (Cho) is usually used as a marker of cell density and membrane renewal, which can reflect the damaged cholinergic neurons in AD. Creatine (Cr) is related to energy metabolism in the brain, mainly including intracellular Cr and Cr phosphate. Cr can be used as a marker of energy metabolism in the brain, and it is often used as an internal reference to measure the content of other metabolites in many spectral studies (Zhang et al., 2015; Fayed et al., 2017). Occasionally, Cr cannot be used as a reference value (Howe et al., 2003) because of abnormal energy metabolism in some diseases, such as a high malignant tumor. A recent study by Mitolo et al. (2019) found that a combination of the ratio of NAA/myoinositol (ml) and the volume of parahippocampal gyrus could improve the overall accuracy of predicting the conversion to AD 2 years before the occurrence of clinical symptoms. The study by Kuhla et al. (2017) has shown that based on the MRI morphology and MRS-based NAA evaluation, APPswe/PS1dE9 mice showed an increase in Aβ plaques, a loss of neurons, and an impairment of the NAA/Cr ratio, but no brain atrophy. It was found that the change of NAA as a functional marker rather than the change in volume was more obvious.

There are few studies on the cutoff point of brain metabolites related to Chinese patients with MCI. The goal of the present study is to explore the relationship between brain metabolites and a cognitive function score in Chinese elderly patients with MCI to provide a reference for clinical diagnosis.

## Methods

All research procedures were conducted in accordance with the Declaration of Helsinki, and the study was registered in http://www.chictr.org.cn. The Clinical Trial Registration Number as ChiCTR-IPR-16009144.

### Subjects

Subjects were selected from the outpatient department, who were admitted from January 2014 to December 2017, of the First Affiliated Hospital of Guangxi University of Traditional Chinese Medicine. The inclusion criteria were as follows: (1) elderly people aged 55–82 with educational experience, (2) the MCI group should meet the MCI diagnostic criteria of the 2006 edition of Chinese guidelines for the diagnosis and treatment of dementia (Tian, 2012), with the chief complaint of memory impairment and abnormal MoCA score (Petersen, 2004; Petersen and Morris, 2005; Lu et al., 2011) (all patients with MCI included in this study belong to the MCI group); and (3) in the normal control (NC) group, the chief complaint of non-cognitive impairment and the total score of MMSE (Chinese version) >27 (Zhang, 2003) was required. The overall cognitive function was normal, and no cerebral infarction and other brain lesions were found on CT or MRI. The exclusion criteria were as follows: (1) non-age group, (2) cardiovascular risk factors were hypertension or diabetes mellitus, patients with a history of acute cardiovascular events, and patients with a history of clinical stroke, (3) ataxia and subjects with a history of ataxia, (4) patients with a history of malignancy, and (5) a current or previous history of smoking, alcohol intake, or drug abuse, and known patients who had used other drugs that may cause a cognitive function change. All participants provided written informed consent before the registration. A total of 136 subjects were recruited, of which 69 of them met the MCI diagnostic criteria were categorized as the MCI group, 67 of them who not have a cognitive decline were categorized as the NC group. All the subjects were provided informed consent according to a protocol approved by the Research Ethics Committee of the First Affiliated Hospital of Guangxi University of Traditional Chinese Medicine.

### MRS Examination

The 3.0T Siemens superconducting MRI system (Siemens, Verio 3.0T) was used to collect the multi-voxel spectrum of the MCI group and the NC group. Point resolved spectroscopy-chemical shift imaging (PRESS-CSI) sequence was used. The repetition time (TR)/the echo time (TE) was 1,700/135 ms, and the frequency was 1,200 Hz. The field of view (FOV) is 160 mm × 160 mm, and a matrix size is 16 × 16. The volume of interest (VOI) is 80 mm × 66 mm × 15 mm for the HIP and 50 mm × 50 mm × 15 mm for the PCG. The voxel size is 10.0 mm × 10.0 mm × 15.0 mm for both of them. The VOI size of the HIP was designed to match the bilateral HIP, which is located in the skull base, to avoid interference from other factors, such as skull and cerebrospinal fluid. An automatic prescanning program was used to adjust the gain of the voxel, receive/transmit, for semiautomatic shimming, weak water suppression, full width at half maximum (FWHM) <25 Hz, and water suppression level >95%. After the scanning, a series of post-processing steps are used to obtain the data of MRS, including water reference processing, filter, zero filling, Fourier transformation, frequency shift correction, baseline correction, phase correction, and curve fitting. The metabolic chemical shift was 2.02, 3.03, and 3.22 ppm for NAA, total Cr (tCr), and Cho, respectively. Considering tCr as an internal parameter, the ratio of NAA/tCr and the ratio of Cho/Cr was calculated. MMSE (Chinese version) and MoCA (Beijing version) were evaluated by the same clinician before an MRS examination in the MCI group and the NC group.

### Neuropsychological Assessment

Cognitive assessments include MMSE (Chinese version) (Zhang, 2003) and MoCA (Beijing version). The MMSE consists of several tasks, testing direction, memory, attention, calculation, language (naming, repetition, auditory comprehension, reading, and writing), and visual special ability, and the highest total score is 30. The MoCA tests eight cognitive areas: (1) visual spatial ability, (2) attention and concentration, (3) executive function, (4) immediate and delayed memory, (5) language, (6) abstract thinking, (7) calculation, (8) orientation, and the highest total score is 30. If the education years of the subjects are <12 years, the measured total score is the test score plus one point. All tests were administered and graded by professionals trained in neuropsychological testing.

### Data Analysis

SPSS 23.0 software was used for data analysis, and descriptive statistics in the form of mean and SD (mean ± SD) were used. The independent-samples *t*-test was used to compare the two groups of quantitative variables. Spearman's correlation coefficient was used to evaluate the correlation between the cognitive score and the brain metabolite level. The receiver operating characteristic (ROC) curve of brain metabolites were drawn, the best boundary point of related brain metabolites of patients with MCI according to the maximum value of the Youden Index (YDI) was predicted, and the sensitivity and specificity with *p* < 0.05 as the difference was calculated.

## Results

### Sociodemographic and Cognitive Assessment

The final sample included 136 participants, including 69 patients with MCI and 67 NCs. The score of sex, age, education, MMSE (Chinese version), and MoCA (Beijing version) of the two groups is shown in Table 1. Compared with the NC group, cognitive scores of the MCI group decreased significantly.

**Table 1:** Descriptive data for the general characteristics (*N* = 136).

**Variable**	**MCI (*n* = 69)**	**NC (*n* = 67)**	***p*-value**	***t-*value**
Sex (% male)	20 (29.0%)	25 (37.3%)	0.722	
Age (y)	64.59 ± 6.66	64.76 ± 5.73	0.876	−0.157
Education level (y)	10.78 ± 2.61	11.85 ± 3.04	0.030	−2.199

*Data are expressed as mean ± SD (range) except where frequencies are used for categorical data*.

### MRS and Cognitive Scores Results

Magnetic resonance spectroscopy in the MCI group was compared with that in the NC group: there were differences in the value of NAA/tCr in the left HIP, the value of NAA/tCr in the right HIP, the value of Cho/tCr in the right HIP, the value of NAA/tCr in the left PCG, and the value of NAA/tCr in the right PCG (*p* < 0.01). However, there were no significant differences in the value of Cho/tCr in the left HIP, the value of Cho/tCr in the left PCG, and the value of Cho/tCr in the right PCG in the MCI group compared with those in the NC group (*p* > 0.05, Table 2).

**Table 2:** MRS test results and cognitive scale scores by diagnostic group (*N* = 136).

**Variable**	**MCI (*n* = 69)**	**NC (*n* = 67)**	***P*-value**	***t*-value**
MMSE	25.87 ± 1.07	29.13 ± 0.76	<0.001	−20.593
MoCA	21.61 ± 2.76	26.03 ± 2.01	<0.001	−10.703
HIP.L NAA/tCr	1.10 ± 0.21	1.38 ± 0.23	<0.001	−7.267
HIP.L Cho/tCr	0.96 ± 0.23	1.00 ± 0.18	0.275	−1.096
HIP.R NAA/tCr	1.09 ± 0.16	1.36 ± 0.25	<0.001	−7.545
HIP.R Cho/tCr	0.90 ± 0.19	1.04 ± 0.19	<0.001	−4.614
PCG.L NAA/tCr	1.87 ± 0.22	2.07 ± 0.21	<0.001	−5.346
PCG.L Cho/tCr	1.02 ± 0.25	1.07 ± 0.18	0.249	−1.158
PCG.R NAA/tCr	1.83 ± 0.25	2.00 ± 0.24	<0.001	−4.088
PCG.R Cho/tCr	1.02 ± 0.20	1.07 ± 0.22	0.184	−1.335

*Data are expressed as mean ± SD (range) except where frequencies are used for categorical data*.

### Correlation Coefficient

The correlation coefficient between cognitive scores and the ratio of NAA/tCr or Cho/tCr ranged from 0.18 to 0.56 (*p* < 0.05), whereas the correlation coefficient between cognitive scores and the ratio of NAA/tCr was 0.49–0.56 in the bilateral HIP (*p* < 0.01, Table 3).

**Table 3:** The correlation coefficient between cognitive scores and MRS at hippocampus (HIP)/posterior cingulate gyrus (PCG) (*N* = 136).

**Variable**	**HIP.L NAA/tCr**	**HIP.L Cho/tCr**	**HIP.R NAA/tCr**	**HIP.R Cho/tCr**	**PCG.L NAA/tCr**	**PCG.L Cho/tCr**	**PCG.R NAA/tCr**	**PCG.R Cho/tCr**	**MoCA**
MMSE	0.49^**^	0.07	0.56^**^	0.39^**^	0.44^**^	0.22^**^	0.37^**^	0.16	0.72^**^
MoCA	0.49^**^	0.08	0.52^**^	0.28^**^	0.36^**^	0.24^**^	0.34^**^	0.18^*^	1.00

** *Correlation is significant at the 0.01 level (two-tailed).*

* *Correlation is significant at the 0.05 level (two-tailed)*.

### ROC Curve

**Figure 1:**
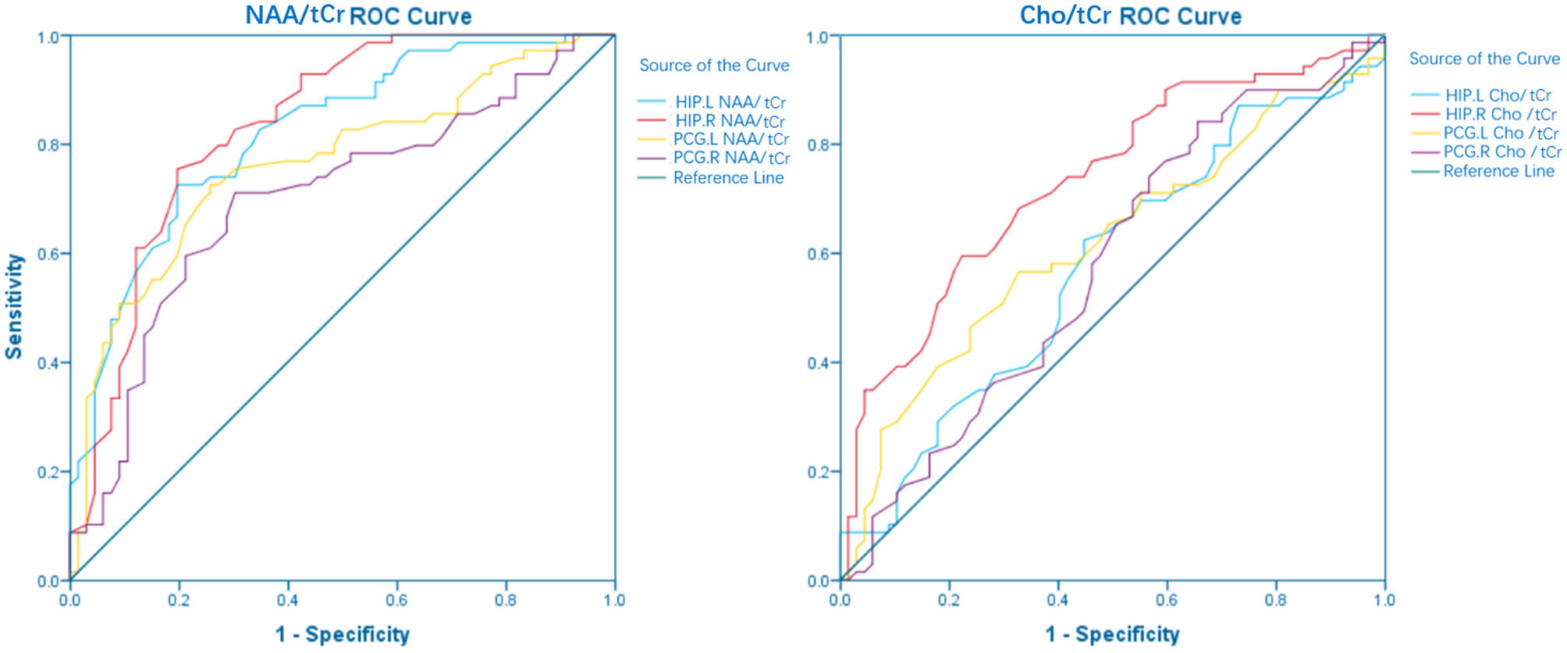
Area under the receiver operating characteristic (ROC) curve and confidence interval (CI): hippocampus left (HIP.L) NAA/total creatinine (tCr): 0.819 (95% CI: 0.749–0.889) *p* < 0.001, HIP right (HIP.R) NAA/tCr: 0.834 (95% CI: 0.765–0.903) *p* < 0.001, posterior cingulate gyrus left (PCG.L) NAA/creatine (Cr): 0.764 (95% CI: 0.683–0.846) *p* < 0.001, PCG right (PCG.R) NAA/tCr: 0.698 (95% CI: 0.608–0.788) *p* < 0.001, HIP.L choline (Cho)/tCr: 0.574 (95% CI: 0.477–0.670) *p* = 0.137, HIP.R Cho/tCr: 0.731 (95% CI: 0.647–0.816) *p* < 0.001, PCG.L Cho/tCr: 0.617 (95% CI: 0.522–0.712) *p* = 0.019, PCG.R Cho/tCr: 0.576 (95% CI: 0.479–0.672) *p* = 0.128.

The larger the area under the ROC curve (AUC), the better it is at indicating a higher diagnostic value. According to Figure 1, NAA/tCr is more sensitive than Cho/tCr, while the curve of the bilateral HIP is more sensitive than that of the bilateral PCG, and the AUC under the NAA/tCr curve of the bilateral HIP is larger. The value of NAA/tCr was the highest in the right HIP, the AUC is 0.834, the area [standard error (SE)] is 0.035 (*p* < 0.001), 95% confidence interval (CI) is 0.765–0.903, and then in the left HIP, the values of AUC is 0.819, the area (SE) is 0.036 (*p* < 0.001), 95% CI is 0.749–0.889 (Table 4 and Figure 1).

**Table 4:** The area under the receiver operating characteristic (ROC) curve.

**Variable**	**AUC**	**Std. Error^**a**^**	***p*-value^**b**^**
HIP.L NAA/tCr	0.819	0.036	<0.001
HIP.L Cho/tCr	0.574	0.049	0.137
HIP.R NAA/tCr	0.834	0.035	<0.001
HIP.R Cho/tCr	0.731	0.043	<0.001
PCG.L NAA/tCr	0.764	0.041	<0.001
PCG.L Cho/tCr	0.617	0.048	0.019
PCG.R NAA/tCr	0.698	0.046	<0.001
PCG.R Cho/tCr	0.576	0.049	0.128

a *Under the non-parametric assumption.*

b *Null hypothesis: true area = 0.5*.

### Cutoff Value

In order to further analyze the discrimination function of different cutoff values, according to the statistical results of the ROC curve, sensitivity, specificity, and the YDI of each cutoff value of NAA/tCr in the bilateral HIP were calculated (Table 5). The YDI of NAA/tCr in the left HIP and the right HIP was maximum at 0.528 and 0.557, and the corresponding optimal cutoff value of the left and right HIP was 1.195 and 1.19. When the NAA/tCr value of the left HIP and the right HIP is <1.19, we suggest that MCI might have occurred. According to this cutoff point, patients with MCI in the elderly could be screened.

**Table 5:** Sensitivity, specificity, and the Youden Index (YDI) corresponding to each point of the ROC curve in the left HIP and the right HIP.

**HIP.L NAA/tCr**	**Sensitivity**	**Specificity**	**Youden index**	**HIP.R NAA/tCr**	**Sensitivity**	**Specificity**	**Youden index**
0.8	0.072	1	0.072	0.87	0.087	1	0.087
0.88	0.159	1	0.159	0.935	0.203	0.955	0.158
0.935	0.232	0.97	0.202	0.995	0.275	0.924	0.199
1	0.304	0.955	0.259	1.025	0.333	0.924	0.257
1.06	0.435	0.924	0.359	1.065	0.42	0.894	0.314
1.115	0.493	0.909	0.402	1.105	0.522	0.879	0.401
1.155	0.652	0.818	0.47	1.145	0.609	0.864	0.473
***1.195***	0.725	0.803	***0.528***	***1.19***	0.754	0.803	***0.557***
1.265	0.783	0.682	0.465	1.255	0.826	0.697	0.523
1.305	0.87	0.576	0.446	1.295	0.899	0.576	0.475
1.355	0.884	0.47	0.354	1.34	0.928	0.53	0.458
1.385	0.913	0.439	0.352	1.375	0.986	0.439	0.425
1.44	0.957	0.394	0.351	1.43	1	0.348	0.348
1.64	0.986	0.136	0.122	1.525	1	0.242	0.242
1.725	0.986	0.091	0.077	1.63	1	0.091	0.091
2.99	1	1	0	3.4	1	1	0

*The bold and italics values represent the maximum Youden index value and the corresponding best cutoff value*.

### Cases

**Figure 2:**
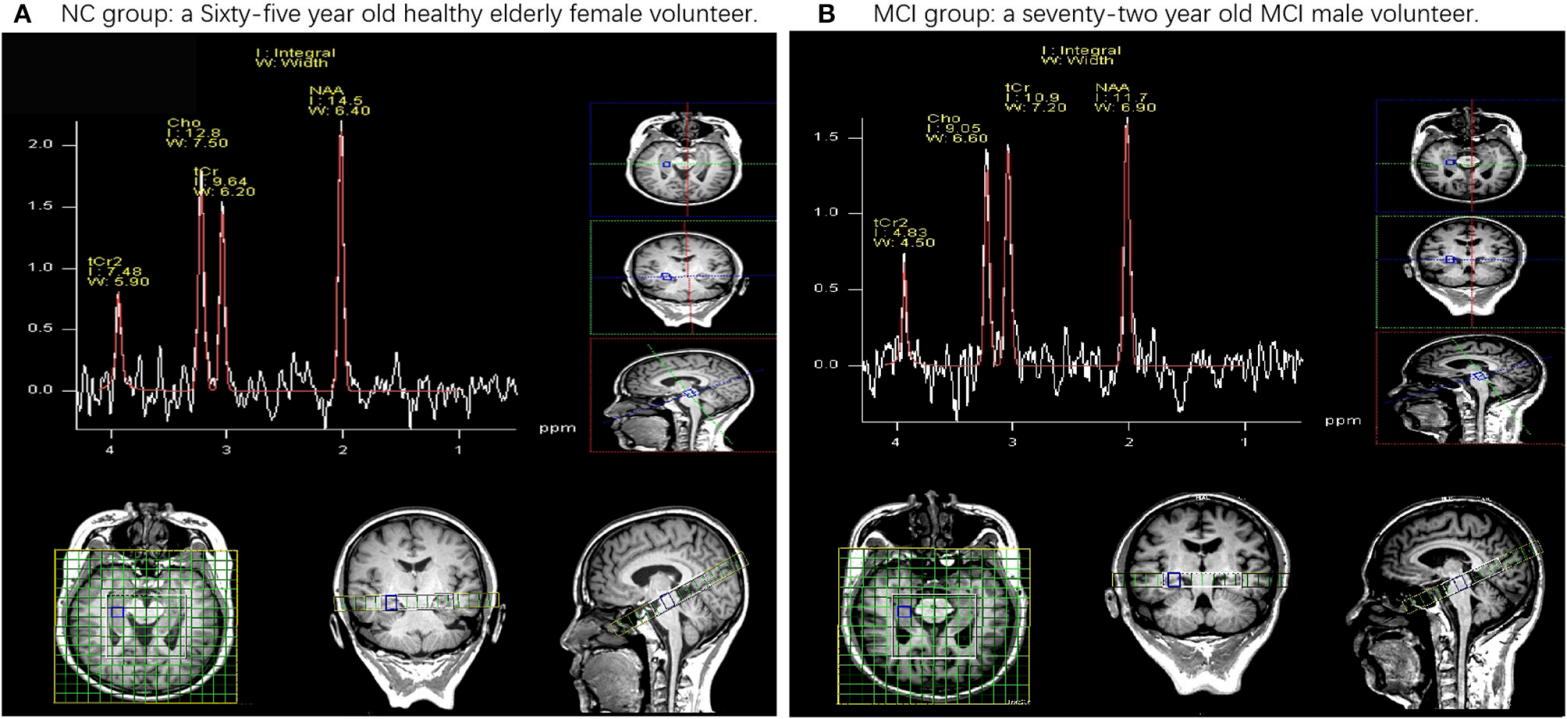
Examples of the data of 1H magnetic resonance spectroscopy (MRS) obtained in healthy elderly female volunteers and mild cognitive impairment (MCI) male volunteers. NAA, *N*-acetyl aspartate; tCr, total creatinine; Cho, choline. **(A)** Demonstrates a 65-year-old healthy elderly female volunteer. These ratios are (NAA/tCr = 1.50, Cho/Cr = 1.33) in the right hip. **(B)** Demonstrates a 72-year-old MCI male volunteer. These ratios are (NAA/tCr = 1.07, Cho/tCr = 0.83) in the right hip.

Figures 2A,B show a demonstration of a sample of the selected cases reported in this study, in which, in each part, there is one case with the normal right HIP (A) and one case with MCI with the right HIP (B).

## Discussion

Mild cognitive impairment is a transitional state between the normal cognitive level and AD (Petersen et al., 1999). In 1991, the concept of “mild cognitive impairment” was first used (Flicker et al., 1991). In this study, we investigated a cognitive function between the MCI group and the NC group and detected a difference in brain metabolites between the two groups using MRS.

A cognitive function scale has been used to measure MCI, and the MMSE and MoCA offer reasonably good diagnostic and classification accuracy (David et al., 2013). MoCA is an effective and rapid screening tool for cognitive dysfunction, involving memory, executive function, and attention (Jiang et al., 2018), and has medium specificity and high sensitivity (Mclennan et al., 2011). In this study, the score of MMSE and MoCA in the MCI group was lower than the score of MMSE and MoCA in the NC group.

Magnetic resonance spectroscopy is a method to analyze a series of specific compounds by using the phenomenon of magnetic resonance in the magnetostatic field and chemical shift characteristics in the static magnetic field. In particular, it can be used in the study of biochemical and metabolic changes in the nervous system. At present, 1H-MRS is more widely used (Graaf, 2010; Su et al., 2016) for a non-invasive quantitative evaluation of the local metabolism level of some substances in the brain tissue. More and more evidence shows that NAA decreases in varying degrees during the transformation of normal people with non-cognitive impairment to MCI (Liu et al., 2013) and the transformation of people with MCI to people with AD (Pilatus et al., 2009). The study showed that the NAA/Cr ratio of patients with MCI was lower than that of patients with NC (Liu et al., 2013). In addition, NAA/Cr was decreased in patients with early AD, and NAA/Cr being an index of decrease was beneficial to the diagnosis of AD (Zhou et al., 2008; Loos et al., 2010). Similar to the previous studies, we found that NAA/tCr of the bilateral HIP and the bilateral PCG was decreased in the MCI group as compared with the NC group.

Meanwhile, we found that Cho/tCr in the bilateral PCG of individuals with MCI had no significant difference compared with the NC group. Research showed that the decrease of metabolism in the PCG was an early sign of AD, and it often appeared before a clinical diagnosis (Raj et al., 2012). Zhou et al. (2008) found that there was no change in the Cho/tCr ratio in patients with early AD. In addition, it has been reported that there is no significant change in the Cho/tCr ratio in the PCG of patients with AD (Kizu et al., 2004). An increase in the Cho level of gray matter is related to a decrease in memory function and local brain metabolism. Findings on Cho have been inconsistent in the previous studies (Mielke et al., 2001; Valenzuela and Sachdev, 2001).

In addition, we found that the expression of Cho/tCr in the HIP and the PCG was different, although there was no significant difference in Cho/tCr between the bilateral PCG, and there was a difference in the decrease of Cho/Cr in the right HIP. The HIP plays an important role in memory processing and is considered to be the first involved area in the AD pathological process (Bradley et al., 2002). The HIP is a widely selected functional area in the study of AD and cognitive memory disorders. For subjects with mild memory impairment (MMI) who do not meet the criteria of memory MCI in a study using MRS in 3T of the bilateral HIP, the decrease of NAA/Cr in the right HIP was more obvious, which suggested that the brain region might have early changes (Caserta et al., 2008). The backmost part of the posterior cingulate cortex (PCC) is usually affected by neurodegenerative diseases. The reduction of metabolism in PCG is an early sign of AD and often occurs before clinical diagnosis (Raj et al., 2012). A recent study shows that alterations in the NAA/ml ratio in PCC show high accuracy in predicting the progression to AD (Mitolo et al., 2019); however, Cho was not determined in this study. Kantarci et al. (2013) reported that quantitative MRI and 1H-MRS, especially the volume of the HIP and the NAA/Cr of the PCC, were good predictors of cognition function, but they did not measure Cho. According to the results of this study, we speculated that, in the development of MCI, the change of Cho in the HIP is earlier than that in the PCG, and a specific change needs further study. In addition, we considered that the difference of Cho/tCr in the bilateral HIP may be related to the right-handedness of patients with MCI.

A prospective study found that the ratio of NAA/Cr in the PCG and the left occipital lobe showed high sensitivity and specificity for predicting the progression of AD in MCI, with the values of sensitivity being 0.82 and 0.72, respectively, and the values of specificity being 0.78 and 0.69, respectively (Fayed et al., 2008). Similarly, Modrego et al. (2011) reported that, when the ratio of NAA/Cr in the middle and the rear of bilateral parietal lobes was ≤ 1.43, the accuracy and specificity of predicting the conversion of MCI to possible AD were 0.741 and 0.837, respectively, when included in the APOE genotype and memory tests for analysis, where the accuracy was higher. Because of the different choices, such as brain function area and race, the cutoff point of NAA/Cr may be different. In this study for the elderly in China, we found that the sensitivity of NAA/tCr to cognitive impairment was higher than that of Cho/tCr. The cutoff point of the ratio of NAA/tCr in the left HIP to distinguish a normal cognitive impairment from MCI was 1.195. Sensitivity, specificity, and the YDI were 0.725, 0.803, and 0.528, respectively. In the right HIP, the cutoff point was 1.19. Sensitivity, specificity, and the YDI were 0.754, 0.803, and 0.557, respectively.

## Conclusion

Based on the study of the metabolic characteristics of local biochemical substances in the HIP of patients with MCI, we suggest that NAA/tCr <1.19 in the bilateral HIP should be used as an evaluation index, combined with the cognitive tests in the differential diagnosis of MCI to provide a reference for the diagnosis of patients with MCI. Because we use long TE, the results might be biased since the T2 relaxation time between the cohorts is unknown, which is the shortcoming of our research. We will optimize it in future research.

## Data Availability Statement

The raw data supporting the conclusions of this article will be made available by the authors, without undue reservation.

## Ethics Statement

The studies involving human participants were reviewed and approved by First Affiliated Hospital of Guangxi University of Traditional Chinese Medicine. The patients/participants provided their written informed consent to participate in this study.

## Author Contributions

LZ, WM, and XD designed the study. LZ, WM, JT, JS, BY, and XN performed the experiments. CL, YW, GD, DD, and SC analyzed and interpreted the data. LZ, XD, and WM wrote the manuscript. All authors read the manuscript, approved the final manuscript, and have made substantial contributions to the manuscript.

## Conflict of Interest

The authors declare that the research was conducted in the absence of any commercial or financial relationships that could be construed as a potential conflict of interest.
