# Corrigendum: A Pilot Study on the Cutoff Value of Related Brain Metabolite in Chinese Elderly Patients With Mild Cognitive Impairment Using MRS

**DOI:** 10.3389/fnagi.2021.759482

**Published:** 2021-10-12

**Authors:** Lihua Zhao, Jinlong Teng, Wei Mai, Jiahui Su, Bihan Yu, Xiucheng Nong, Chong Li, Yichen Wei, Gaoxiong Duan, Xiangming Deng, Demao Deng, Shangjie Chen

**Affiliations:** ^1^Department of Acupuncture, First Affiliated Hospital, Guangxi University of Chinese Medicine, Nanning, China; ^2^Department of Radiology, First Affiliated Hospital, Guangxi University of Chinese Medicine, Nanning, China; ^3^Department of Rehabilitation, Bao'an Hospital, Southern Medical University, Shenzhen, China

**Keywords:** N-acetyl aspartate, creatine, choline hippocampus, posterior cingulate gyrus, mild cognitive impairment

In the original article, there were mistakes in Table 1 and Table 2 as published. Due to the negligence of the inputter, the standard deviation of MMSE in MCI group in Table 1 was wrong. These values should instead be 25.87 ± 1.07. The MMSE and MoCA data should also be removed from Table 1, and instead placed in Table 2. Additionally, Table 2 incorrectly showed the data calculation results of 64 patients with MCI and 30 patients with NC. The corrected Table 2 shows the 69 patients with MCI and 67 patients with NC, and the N value is 136. The corrected Tables 1 and 2 appear below.

**Table 1:** Descriptive data for the general characteristics (*N* = 136).

**Variable**	**MCI (*n* = 69)**	**NC (*n* = 67)**	***p*-value**	***t-*value**
Sex (% male)	20 (29.0%)	25 (37.3%)	0.722	
Age (y)	64.59 ± 6.66	64.76 ± 5.73	0.876	−0.157
Education level (y)	10.78 ± 2.61	11.85 ± 3.04	0.030	−2.199

*Data are expressed as mean ± SD (range) except where frequencies are used for categorical data*.

**Table 2:** MRS test results and cognitive scale scores by diagnostic group (*N* = 136).

**Variable**	**MCI (*n* = 69)**	**NC (*n* = 67)**	***P*-value**	***t*-value**
MMSE	25.87 ± 1.07	29.13 ± 0.76	<0.001	−20.593
MoCA	21.61 ± 2.76	26.03 ± 2.01	<0.001	−10.703
HIP.L NAA/tCr	1.10 ± 0.21	1.38 ± 0.23	<0.001	−7.267
HIP.L Cho/tCr	0.96 ± 0.23	1.00 ± 0.18	0.275	−1.096
HIP.R NAA/tCr	1.09 ± 0.16	1.36 ± 0.25	<0.001	−7.545
HIP.R Cho/tCr	0.90 ± 0.19	1.04 ± 0.19	<0.001	−4.614
PCG.L NAA/tCr	1.87 ± 0.22	2.07 ± 0.21	<0.001	−5.346
PCG.L Cho/tCr	1.02 ± 0.25	1.07 ± 0.18	0.249	−1.158
PCG.R NAA/tCr	1.83 ± 0.25	2.00 ± 0.24	<0.001	−4.088
PCG.R Cho/tCr	1.02 ± 0.20	1.07 ± 0.22	0.184	−1.335

*Data are expressed as mean ± SD (range) except where frequencies are used for categorical data*.

The authors apologize for this error and state that this does not change the scientific conclusions of the article in any way. The original article has been updated.

## Publisher's Note

All claims expressed in this article are solely those of the authors and do not necessarily represent those of their affiliated organizations, or those of the publisher, the editors and the reviewers. Any product that may be evaluated in this article, or claim that may be made by its manufacturer, is not guaranteed or endorsed by the publisher.

